# The Role of the Gut Microbiome in Diabetes and Obesity-Related Kidney Disease

**DOI:** 10.3390/ijms22179641

**Published:** 2021-09-06

**Authors:** Amgad Zaky, Sarah J. Glastras, May Y. W. Wong, Carol A. Pollock, Sonia Saad

**Affiliations:** 1 Renal Research Laboratory, Kolling Institute of Medical Research, University of Sydney, Sydney, NSW 2065, Australia; amgad.zaky@sydney.edu.au (A.Z.); sarah.glastras@sydney.edu.au (S.J.G.); wong.may.yw@gmail.com (M.Y.W.W.); carol.pollock@sydney.edu.au (C.A.P.); 2 Royal North Shore Hospital, St. Leonards, NSW 2065, Australia

**Keywords:** microbiota, diabetes, obesity, kidney disease

## Abstract

Diabetic kidney disease (DKD) is a progressive disorder, which is increasing globally in prevalence due to the increased incidence of obesity and diabetes mellitus. Despite optimal clinical management, a significant number of patients with diabetes develop DKD. Hence, hitherto unrecognized factors are likely to be involved in the initiation and progression of DKD. An extensive number of studies have demonstrated the role of microbiota in health and disease. Dysregulation in the microbiota resulting in a deficiency of short chain fatty acids (SCFAs) such as propionate, acetate, and butyrate, by-products of healthy gut microbiota metabolism, have been demonstrated in obesity, type 1 and type 2 diabetes. However, it is not clear to date whether such changes in the microbiota are causative or merely associated with the diseases. It is also not clear which microbiota have protective effects on humans. Few studies have investigated the centrality of reduced SCFA in DKD development and progression or the potential therapeutic effects of supplemental SCFAs on insulin resistance, inflammation, and metabolic changes. SCFA receptors are expressed in the kidneys, and emerging data have demonstrated that intestinal dysbiosis activates the renal renin-angiotensin system, which contributes to the development of DKD. In this review, we will summarize the complex relationship between the gut microbiota and the kidney, examine the evidence for the role of gut dysbiosis in diabetes and obesity-related kidney disease, and explore the mechanisms involved. In addition, we will describe the role of potential therapies that modulate the gut microbiota to prevent or reduce kidney disease progression.

## 1. Introduction

Diabetic kidney disease (DKD) is a devastating complication of both type 1 and type 2 diabetes mellitus (T1D and T2D), predicted to affect around 40% of patients with diabetes by 2045 [1,2]. DKD significantly increases the risk of developing cardiovascular disease and end-stage kidney disease (ESKD), which ultimately necessitates dialysis or kidney transplantation [3,4]. The global incidence of ESKD continues to increase by 6–12% annually, driven by the increasing prevalence of obesity and T2D [5]. 

The microbiome has 150 times more genes than the human genome [6]. The microbiota is established from birth and shaped during the first three years of life [7,8]. Gender, age, family history, ethnicity, dietary preference, geographical areas of living, use of probiotics, prebiotics, and antibiotics, are all factors which influence the gut microbiota composition [9,10,11]. The gut microbiota is important in maintaining the gastrointestinal and immune functions through the digestion and fermentation of nutrients as well as electrolyte and mineral absorption [9,12]. It collects signals from the surrounding environment and nutrients to produce metabolites working symbiotically with the immune system. Such signals boost the defense against different pathogens resulting in health promotion [13,14]. 

The gut microbiota plays an important role in physiology and disease state, including obesity, diabetes, asthma, allergy, cancer, cardiovascular disease, and aging, and more recently, gut dysbiosis has been implicated in DKD [15,16,17,18,19]. Diabetes and obesity, which are common in patients with T2D, are associated with alterations in the gut microbiota, and recent studies suggest that DKD initiation and progression are associated with an altered gut microbial ecology or dysbiosis [20,21]. This review will summarise the evidence for the role of gut dysbiosis in diabetes, obesity, and DKD, raising the possibility that the gut microbiota may be a potential target to prevent and reduce the progression of DKD.

## 2. Gut Microbiota Community

The gut microbiota is a dynamic community of microorganisms made up of 100 trillion microbes living within the gastrointestinal system of the host organism. Bacteria make up the majority of the microorganisms, though viruses and fungi also contribute [15,22], accounting for 1.5 to 2 kg of human body weight [9,23]. Six phyla are dominant in the gut microbiota, including Fusobacteria, Firmicutes, Proteobacteria, Verrucomicrobia, Actinobacteria, and Bacteroidetes, with Firmicutes and Bacteroidetes representing around 90% of the gut microbiota [24,25]. 

Fusobacterium are generally considered pathogenic since Fusobacterium strains cause several human diseases. Firmicutes and proteobacteria are also considered harmful bacteria due to their negative influence on glucose and fat metabolism. However, Verrucomicrobia, actinobacteria, and Bacteroidetes are considered ‘good’ bacteria due to their contribution to intestinal health and glucose homeostasis [26], host resistance to infective disease [27], and the production of favorable metabolites such as short-chain fatty acids (SCFAs), known to reduce inflammation [28].

The Bacteroidetes and Firmicutes phyla normally contribute the most to gut microbiota [29]. *Bacteroides* and *Prevotella* are the two dominant genera of the Bacteroidetes phylum, while *Clostridium*, *Ruminococcus*, *Bacillus*, *Lactobacillus*, and *Enterococcus*, alongside 200 other genera, form the phylum of Firmicutes [25]. The Clostridia class of the *Clostridium* genus forms 95% of the Firmicutes phylum [29].

## 3. Gut Microbiota By-Products and Functions

### 3.1. Short Chain Fatty Acids—The By-Products of Microbial Metabolism

Polysaccharide fermentation, induced by gut microbiota, produces short chain fatty acids (SCFA), which are utilized by colon epithelial cells as nutrition. Propionate, acetate, and butyrate are the main SCFAs produced by the bacterial fermentation of dietary fiber in the gut [30]. SCFAs provide around 6–10% of the body’s total daily energy [31,32]. The majority of SCFAs are absorbed in the colon (90–95%), while the rest (5–10%) are secreted within the stool. About 60% of SCFA absorption takes place via epithelial membrane diffusion, whilst the remainder is absorbed by active cell transportation, specifically monocarboxylate transporters in colonocytes [33,34]. Those which are not metabolized by colonocytes (mainly butyric acid) are transported through the portal circulation and metabolized in the liver before they reach the systemic circulation. However, the distal colon, where most of the gut microbiota reside, bypasses the portal circulation, allowing systemic access [30,35]. Hence, SCFAs produced by the microbiota can be present in portal, hepatic, peripheral blood, and feces [35,36,37]. Low levels of SCFAs in the blood and gastrointestinal system are implicated in diabetes and inflammatory disease [38,39]. 

### 3.2. Short Chain Fatty Acid Interaction with End-Organ Receptors

**Figure 1:**
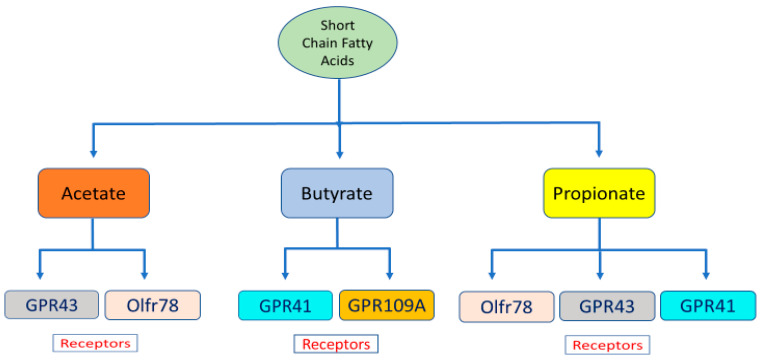
SCFA receptors and their relationship with different types of SCFAs.

The SCFAs produced by the gut microbiota are absorbed into the bloodstream and reach distant tissues such as the liver, adipose tissue, and kidneys after their uptake by G protein-coupled receptors (GPRs), including GPR41, GPR43, GPR109A, and Olf78. SCFAs differentially activate GPRs; propionate activates GPR41 and GPR43, acetate activates GPR43, and butyrate activates GPR41 [40] (Figure 1). Through the activation of GPR41 and 43 within adipocytes, epithelial and mononuclear cells, SCFAs initiate adipogenesis, suppress the synthesis of cholesterol and fatty acids in the liver, and regulate obesity in mice [30,41]. In addition, GPR-41 activation improves blood pressure regulation, whereas GPR-43 activation enhances cell immune responses [38].

GPR-109A may have an additional role in limiting inflammation [42]. Animal and human studies have demonstrated an association between GPR109A expression and colon cancer, suggesting it may be a tumor suppressor [38,43,44]. GPR109A activated by SCFAs also inhibits NF-kB activation and pancreatic beta cells inflammation in mice [44]. 

Unlike the other receptors, the Olfr-78 receptor does not respond to butyrate but is more sensitive to propionate and acetate [45]. In addition to its role in olfaction and hormone regulation, Olfr-78 acts as a hypoxia sensor and plays a role in renin secretion and blood pressure regulation [46,47,48]. 

Olfr-78, GPR-43, GPR-41, and GPR-109A are all expressed in the kidney [46,49,50], such that SCFAs can influence kidney health. Olfr-78 is expressed in the renal juxtaglomerular apparatus and smooth muscle cells of small resistance vessels [46]; its human orthologue (hOR51E2) is expressed in the human kidney, as well as in multiple other tissues such as heart and skeletal muscle [49]. GPR-43 is expressed in human embryonic kidney cells [50]. GPR-41 is also expressed in renal arteries and smooth muscle cells, suggesting that the biological role of these receptors possibly extends beyond their role in regulating the release of peptide hormone from enteroendocrine cells in the gut to include modulation of kidney health [51]. Finally, increased expression of GPR109A in podocytes shows a protective effect against proteinuria in an animal model of renal injury by stabilizing glomerular basement membrane podocytes and attenuating glomerulosclerosis and renal inflammation [52]. Hence, SCFAs produced by the gut microbiota have a potential role in kidney health and disease. 

Multiple studies have demonstrated the role of SCFAs in gene regulation of anti-inflammatory processes both in vitro and in vivo [53]. SCFAs are implicated in energy metabolism and are known to affect lipid, glucose, and cholesterol metabolism and improve insulin sensitivity; thus, supporting the role of microbiota in glycemic control [54,55]. The effect of SCFAs on gene transcription can occur via epigenetic regulation—heritable phenotype changes that do not involve alterations in the DNA sequence [56,57]. In intestinal epithelial cells, propionate and butyrate have been shown to inhibit the activity of histone deacetylase enzymes (HDAC3 and HDAC1), which in turn increase histone acetylation. The inhibition of HDACs by SCFAs can decrease histone compactness, NF-κB (nuclear factor κB) and myogenic antigen differentiation, colonocyte P53 expression, and nuclear factor of activated T cell (NFAT) production [38,58,59]. Microbiota-derived SCFAs were recently shown to promote the post-translational modification of histones in the colon through histone deacetylation. Epigenetic modifications due to SCFAs have also been implicated in the development of diabetes and chronic kidney disease [60,61]. 

## 4. Dysbiosis in Diabetes, Obesity, and Chronic Kidney Disease

### 4.1. Role of the Gut Microbiota in Type 1 Diabetes

The incidence of T1D has steeply risen in recent decades in both developed and developing countries; genetic factors are inadequate to explain the increased incidence, leading to investigators searching for alternate explanations. 

T1D is an autoimmune disease. It is known that lipopolysaccharide (LPS) plays a significant role in increasing the level of pro-inflammatory cytokines via toll-like receptors (TLRs) [62,63,64], thereby playing a possible role in causing pancreatic beta-cell failure in susceptible individuals. LPS is a major component of the outer membrane of Gram-negative bacterial species, and LPS can be derived from microbiota in the gut [62,65]. A direct causal relationship between gut dysbiosis and the development of T1D is yet to be established. However, mounting evidence strongly suggests a link. There is increasing evidence to suggest that the gut microbiota plays an integral role in this altered trajectory from early life in both animal and human studies (Table 1 and Table 2, respectively). Multiple studies have demonstrated that patients with T1D have distinctly different gut microbiota, in comparison with healthy subjects, characterized by a decreased Firmicutes (Gram-positive): Bacteroidetes (Gram-negative) ratio [66,67,68,69,70]. *Bacteroides* were more prevalent in patients with T1D in association with an increase in inflammatory cytokine, interleukin (IL)-6, and poor glycemic control [71]. *Bacteroides ovatus* encompassed around 24% of the total increase in the phylum Bacteroidetes [70]. A higher amount of Bacteroidetes in patients with T1D correlated with increased toll-like receptor (TLR)-4, TLR-2 levels [66], and anti-islet cell autoantibodies [67]. *Faecalibacterium* levels are also negatively correlated with glycated hemoglobin (HbA1c) [67]. In addition, increased Bacteroidetes and lower levels of Faecalibacterium were also demonstrated in patients with MODY2 (maturity-onset diabetes of the young 2) [72]. It is worth noting that Pellegrini et al. showed an increased Firmicutes: Bacteroidetes ratio in a small cohort of patients with T1D compared to the control [73]. The increase in Firmicutes was mostly related to the abundant levels of Bacilli class and *Streptococcus* genus. It is not clear whether this discrepancy is due to geographical differences, small sample sizes, or the analytic data techniques used. 

Animal and human studies demonstrated reduced bacterial diversity and stability over time in autoimmune individuals and in patients with T1D [70,72,77]. However, children who will develop autoimmunity have a microbiota that is less diverse and stable [70]. Reduction in the levels of beneficial anaerobic bacteria (*Bifidobacterium* genus) and an increase in the levels of *Candida albicans* and *Enterobacteriaceae* (other than *Escherichia coli*) species were demonstrated in newly diagnosed T1D vs. control [84]. Patterson et al. demonstrated that T1D-onset was associated with an increase in the Bacteroidetes: Firmicutes ratio [77]. Rats that were protected from diabetes had a lower amount of Bacteroides [80] and modulation of microbiota by antibiotics was able to reduce the incidence of T1D and delay its onset [80].

Children with T1D had reduced levels of *Blautia coccoides-Eubacterium rectal group* (involved in butyrate production and maintenance of gut integrity), whereas healthy children had more levels of butyrate-producing species such as *Clostridium* clusters IV and XIVa compared to children with T1D [83]. Increased *Clostridium*, *Bacteroides*, and *Veillonella* genera and reduced *Lactobacillus*, *Prevotella*, and *Bifidobacterium* genera were also demonstrated in children with T1D [68]. Some studies demonstrated that alterations in the gut microbiota might precede the development of T1D [[69,70,74,80]. Indeed, Firmicutes declined, and Bacteroidetes increased in the gut microbiota as children develop T1D, and hence a lower ratio of Firmicutes: Bacteroidetes level was detected in normal children compared to children who become autoimmune [70]. Brown et al. demonstrated that the lactate-producing bacteria (*Lactobacillus*, *Lactococcus*, *Streptococcus*, and *Bifidobacterium*) were less abundant in autoimmunity. Interestingly, increased *Lactobacillus* and *Bifidobacterium* were associated with late-stage T1D progression [77]. Increased butyrate-producing bacteria and mucin-degrading bacteria were shown in the control compared to autoimmune groups. The reduction of mucin-producing bacteria in autoimmunity can contribute to the gut immune imbalance and reduction of gut integrity in the population. Autoimmune microbiota instability and alteration in the ratio of Firmicutes: Bacteroidetes within the first 6 months after birth may be an indication of future development of autoimmunity, before the detection of serum antibodies, and has potential for early diagnoses. 

### 4.2. Role of the Gut Microbiota in Type 2 Diabetes 

Compared with the available evidence in T1D, there is less information about the role of the gut microbiota and the development and progression of T2D (Table 3 and Table 4).

Recent animal studies suggest a relationship between gut dysbiosis and insulin resistance that underpins T2D [97], involving mechanisms that include increased endotoxemia, bowel permeability changes, bile acids interaction, and changes in brown fat distribution [9]. Modification of the gut microbiota by probiotics and/or prebiotics appears to promote glucose homeostasis and improved insulin resistance [97]. Dao et al. showed the gut profusion of *Akkermansia muciniphila* bacteria is directly associated with improvements in glucose homeostasis in humans with early T2D [98].

Moroti et al. demonstrated that administration of symbiotic shakes containing *Bifidobacterium bifidum* and *lactobacillus acidophilus* to patients with T2D decreased fasting blood glucose levels [99]. Alterations of the gut microbiota composition to that resembling the healthy controls had a positive impact on T2D-associated diseases, such as diabetic foot ulcers and diabetic retinopathy [100,101]. 

Overall, as in T1D, a decrease in gut microbe diversity and richness was demonstrated in patients with type 2 diabetes compared to healthy controls [89,90]. Pre-diabetic and diabetic groups had specific metabolic characteristics and gut bacteria [90]. Patients with T2D had reduced levels of Butyrate-producing bacteria such as *Bifidobacterium*, *Akkermansia*, and *Faecalibacterium* [89,92,94] and reduced levels of the Firmicutes phylum, and *Clostridiaceae* and *Peptostreptococcaceaea* families compared to healthy controls [91]. Significant reduction in the *Blautia* genus was also demonstrated in patients with T2D and this negatively correlated with HbA1c and glucose levels [93]. Using an animal model of T2D, Everard et al. demonstrated that *Akkermansia (A) muciniphila* is reduced in mice with T2D, and administration of *A. muciniphila* increased the levels of endocannabinoids and controlled inflammation [88].

Increased Bacteroidetes and decreased Firmicutes levels were demonstrated at the onset of T2D [90]. Patients with T2D also had increased levels of Actinobacteria and Bacteroidetes phyla [91] and *Lactobacillus* [92,94]. Reduced frequency of *Lactobacillaceae* in the T2D group is associated with GLP-1 resistance [87]. Hence, Alterations in the microbiota levels can improve GLP-1-based therapies. The Bacteroidetes to Firmicutes ratio and *Bacteroides-Prevotella to Blautia coccoides -Eubacterium* rectal were also increased in patients with T2D, and this was shown to positively correlate with plasma glucose levels [95]. The levels of *Clostridia* in the stool were decreased in patients with T2D [95]. It is interesting to note that the reduced levels of *Bifidobacteria* and Firmicutes: Bacteroidetes ratio were similarly observed in patients with T1D. Human studies have also demonstrated that patients with T2D have increased levels of *Dora* genus (from the family *Lachnospiraceae*), which is known to be involved in chronic inflammation, and it is proposed that such levels could identify patients at high risk of T2D [89]. Collectively, these studies demonstrated an important role of the microbiota on T2D development. In addition, Bilen et al. demonstrated that *Staphylococcus aureus* and *Staphylococcus epidermidis* were increased in conjunctival flora from patients with T2D compared to control [96]. Dysbiosis in the microbiota seen in animals with T2D was associated with increased resistance to chemotherapy [85]. 

## 5. Role of the Gut Microbiota in Obesity

There is a bidirectional relationship between gut dysbiosis and obesity. Imbalanced gut microbiota is linked to the development of obesity through the modulation of energy homeostasis, autonomous contribution to fat accumulation, and decreased activity of lipoprotein lipase enzyme [9]. Feeding germ-free rodents a high-fat diet (HFD) to induce obesity produces changes in the gut microbiota, such as increased Firmicutes and Proteobacteria bacteria together with decreased Bacteroidetes bacteria [102]. Animals and human studies support the role of the gut microbiota in obesity (Table 5 and Table 6). 

Briefly, microbial diversity was reduced in obese individuals compared to the healthy controls [108,109]. Significant increases in the levels of Proteobacteria phylum [102,109], *Staphylococcus aureus* species [110,111], and *Bacteroides* genus [111] were demonstrated with obesity. Bacteroidetes phylum [102] and the levels of beneficial microbiota such as *Bifidobacterium* genus [109,110,111], anti-inflammatory *Faecalibacterium*, and butyrate-producing *Ruminococcaceae* were reduced due to obesity [109]. Contrary to T1D and T2D, Firmicutes levels were mostly increased in human and mice models of obesity-induced by HFD [102,103,104,108]. However, animal data related to the changes in gut microbiota profiles due to obesity are not consistent and seem to depend on the diet type, and genetic background of the animal used [105]. 

Interestingly, when isolated microbiota from obese animals was transplanted (by oral gavage) into the colon of a germ-free animal, obesity developed after 14 days. In contrast, germ-free animals remained lean after fecal transplantation from a lean animal; although, transplantation of the gut microbiota from a chow-fed germ-exposed mouse to a germ-free mouse led to a 60% body weight gain after two weeks [112]. Similarly, Turnbaugh et al. demonstrated that colonization of germ-free mice with obese microbiota increased total body fat [107], supporting the role of microbiota in the development of obesity. Stool transplantation from telmisartan-treated mice to obese mice attenuated body weight due to HFD [104]. Interestingly, Collado et al. demonstrated an increase in microbial counts during pregnancy. High Bacteroidetes concentrations before pregnancy were associated with excessive weight gain during pregnancy, and the mother’s weight and body mass index correlated with the concentrations of *Clostridium*, Bacteroidetes, and *Staphylococcus* [111]. This suggests that the microbiota composition before the onset of pregnancy and during pregnancy may have an important role in the development of metabolic disease in the mothers and adverse fetal outcomes or obesity in the offspring. Furthermore, a prospective follow-up study of children between the age of 3 months and 7 years demonstrated that children who had higher levels of *Bifidobacteria* maintained a normal weight over time, whereas children who had greater numbers of *Staphylococcus aureus* became overweight [110]. These data suggest that gut microbiota composition can be used to identify individuals at risk of obesity and that modulating the microbiota can provide a novel preventative measure or therapeutic option for weight management.

The mechanisms by which the gut microbiota interacts with obesity are not fully elucidated. The inflammatory responses observed in response to gut bacteria are likely to play a key role. Bacteria produce lipopolysaccharide (LPS), which induces inflammation and oxidative stress by being absorbed by the intestines, so-called metabolic endotoxemia. Lipid A is a structural ingredient of LPS that induces the activation of different pro-inflammatory pathways, leading to increased oxidative stress by binding to its receptor, TLR4 [113,114]. The low-grade inflammation induced by the pro-inflammatory cytokines such as IL-6 and TNF-α leads to metabolic dysfunction detected in obesity [115]. 

Another obesogenic mechanism is the inhibition of the fasting-induced adipose factor (FIAF) expressed in adipose tissue, liver, and intestines. FIAF suppresses the circulating lipoprotein lipase (LPL) enzyme and regulates the metabolism of triglycerides [116]. As the active LPL enzyme stimulates lipoprotein formation, such as chylomicrons and very low-density lipoprotein from triglycerides, FIAF stimulates the segregation of this active LPL enzyme (dimers) into inactive LPL enzyme (monomers) in mice [117]. Bäckhed et al., in an animal study on obese germ-free FIAF ^−/−^ mice, suggested that gut microbiota regulates the storage of energy by FIAF modulation in the mice, and it is a key factor that regulates the energy harvest from food [112]. The administration of *Bacteroides thetaiotaomicron* and *Methanobrevibacter smithii* diminished FIAF production, which leads to increased activity of lipoprotein lipase enzyme and hence increased triglyceride-derived fatty acids storage in adipose tissue and liver [9,118].

The metabolites derived from the gut microbiota, namely LPS and SCFAs, contribute to host obesity and fat storage [119,120,121]. SCFAs are known to increase leptin release from mouse adipose tissue, resulting in appetite inhibition, metabolic rate enhancement, and reduced obesity in mice and humans [30,122]. GPR-43 deficient mice on a normal diet are obese; however, mice overexpressing GPR-43 in adipose tissue stay lean, even if on a high-fat diet [123]. Thus, demonstrating that GPR-43 is involved in regulating energy utilization. Other animal studies which explored the relationship between gut microbiota and obesity through tryptophan-derived metabolites showed that the dysregulation of tryptophan-derived metabolites results in the development of white adipose tissue and insulin sensitivity, ultimately resulting in obesity [107,124].

## 6. The Role of the Gut Microbiota in Chronic Kidney Disease

There is growing evidence to support the role of gut microbiota in the development and progression of CKD, which may at least partly explain why some people develop progressive disease whilst others are seemingly unaffected. Decreased levels of the beneficial gut microbes, their associated SCFAs, and increased levels of harmful microbes were associated with CKD development [125]. In the context of any form of CKD, there is increased permeability of the intestinal barrier leading to transfer of toxins into the blood circulation; the effect is self-perpetuating in CKD, given the context of impaired renal function, endotoxins accumulate in the blood contributing to persistent systemic inflammation, pro-inflammatory cytokine release, and oxidative stress [125,126,127,128,129,130]. Collectively, these data provide proof of concept for the potential role of microbiota in limiting renal fibrosis.

Recent animal and clinical studies have linked gut dysbiosis with diabetes-related complications, including retinopathy, neuropathic foot ulcer, atherosclerosis, hypertension [100], and CKD (summarised in Table 7 and Table 8). 

As in T1D and T2D, a decrease in microbial diversity was also shown in hypertensive rats vs. controls. However, the Firmicutes: Bacteroidetes ratio was increased in hypertensive rats, and this was associated with reduced acetate and butyrate-producing bacteria [133]. Mice with CKD also had reduced levels of *Lactobacillus*, *Oscillospira*, and unclassified *Ruminococcaceae* genera and increased levels of *Bifidobacterium*, *Turicibacter*, and *Allobaculum* genera, leading to inflammation and renal failure [131]. Using an adenine-induced CKD mice model, Yang et al. demonstrated that nine bacterial genera were enriched in CKD, six of which were reduced with fiber supplements [132]. In addition, Tanida et al. showed a decrease in blood pressure following administration of hypertensive rats with *Lactobacillus johnsonii* species [135]. Furthermore, Kawase et al. showed decreased systolic blood pressure and total cholesterol level and increased HDL after feeding humans and rats with fermented milk containing *Streptococcus thermophilus* and *Lactobacillus casei* compared to the healthy groups which were not administered with fermented milk [136]. Collectively, these studies support the role of gut microbiota in CKD and suggest that modulation of the microbiota can impact CKD development or progression.

There is also mounting evidence in humans demonstrating a link between gut dysbiosis and CKD (Table 8). Vallianou et al. reviewed several human studies of gut microbiota in hypertensive patients, finding a high correlation between high blood pressure, and decreased gut microbial diversity, and serum markers of inflammation. They postulated that gut dysbiosis and inflammation impact the kidney by sympathomimetic changes through changes in plasma metabolite secretion, plasma metabolite retention, and body fluid homeostasis [137]. The specific changes, unique or otherwise, in the context of diabetic kidney disease, have been poorly described within human studies. The imbalance of gut microbiota in CKD occurred both quantitatively and qualitatively and is often accompanied by increased levels of *Lachnospiraceae*, *Enterobacteriaceae*, and certain *Ruminococcaceae*, and a reduction in some species of *Bacteroidaceae*, *Lactobacillus*, *Prevotellaceae*, and *Bifidobacterium* [138].

Reduced bacterial diversity was shown in hypertensive patients and in patients with CKD compared to healthy controls [19,133]. As shown in hypertensive rats, Yang et al. demonstrated that the Firmicutes: Bacteroidetes ratio was also increased in hypertensive patients, and this was associated with reduced acetate and butyrate producing bacteria [133]. The levels of opportunistic pathogens from gamma-proteobacteria were also increased in patients with CKD. However, beneficial microbes like *Roseburia*, *Coprococcus*, and *Ruminococcaceae* were decreased [19]. It is not clear whether renal function affects dysbiosis or vice versa, but Xu et al. have demonstrated that impaired renal function and dysbiosis in the gut microbiota increased the plasma concentration of trimethylamine-N-oxide [19] involved in cardiovascular disease and atherosclerosis [139] and hence increased disease comorbidities/complications.

Patients with mild kidney function have reduced butyrate-producing bacteria *Roseburia* spp. and *Faecalibacterium prausnitzii* compared with healthy individuals [140]. A reduced level of culturable anaerobic bacteria was also seen in the feces of these patients compared to healthy controls [141]. However, an increase in culturable aerobic bacteria (such as *Enterococci* and *Enterobacteria* species) was detected in the feces of patients with CKD who had not started dialysis [142] and in patients with ESKD compared with healthy adults [143].

Peritoneal dialysis and post-transplant patients also had lower bacterial species richness compared with healthy individuals and patients on hemodialysis; interestingly, this was associated with reduced levels of Firmicutes and *Bifidobacteria* and increased levels of Bacteroidetes [145]. Patients with ESKD have abundant levels of bacterial families that possess uricase, urease, indole, and p-cresol-forming enzymes and a lower level of bacterial families with butyrate-forming capacity compared with healthy individuals [144,146]. In addition, they have reduced levels of *Bifidobacterium* and *Lactobacillus* spp. compared with healthy individuals [147]. Overall, the abundance of approximately 190 microbial operational taxonomic units were significantly different when comparing patients with ESKD undergoing hemodialysis and healthy controls [134]. Interestingly, bacterial genera grown in the guts of patients with ESKD (such as *Klebsiella*, *Escherichia*, *Proteus*, *Enterobacter*, and *Pseudomonas *spp.) were also detected in the blood of 20% of the patients. This was associated with increased levels of IL-6, C-reactive protein, and plasma D-lactate [148], which demonstrate that bacterial translocation can occur in patients with ESKD and contribute to microinflammation in this population. 

Although the above strongly supports the role of the microbiota in CKD, it is still unclear whether changes in the microbiota are causative of the disease or occur as a consequence of CKD. It is yet to be established whether the microbiota can be used as predictive markers for CKD progression. The mechanism/s whereby the microbiota can influence the rate of progression of CKD is, to date, not well described. It is known that SCFAs have an important role in autophagy, mitophagy, and oxidative stress [149,150], which we and others have previously demonstrated to be dysregulated in animals with DKD [151,152,153]; SCFAs have been shown to normalize autophagy and improve renal dysfunction in a mice model of acute kidney injury [150]. Furthermore, SCFAs have also been shown to have renoprotective effects in experimentally induced diabetic rats [154]. Infusion of the SCFA butyrate decreases extracellular matrix accumulation associated with podocyte injury in the glomerular basement membrane of the kidney in Gpr109a^-/−^ male mice [52]. Furthermore, butyrate lowers renal inflammation, as indicated by the reduction in pro-inflammation cytokine levels of IL1β and tumor necrosis factor (TNF) α [52]. Recent studies have demonstrated that SCFAs improve blood pressure regulation by activating the two receptors, Olfr-78 and GPR-41 [55,155], which in turn modulate renin secretion and vascular tone [156]. The decrease in systolic blood pressure and total cholesterol levels reduce the risk of ischemic heart and cerebral disease often seen in patients with CKD and potentially reduce CKD progression. 

## 7. Regulation of the Gut Microbiota as a Potential Treatment for Diabetic Kidney Disease 

Glucose-lowering agents are used to treat diabetes and restore glucose homeostasis. Despite the large number of glucose-lowering agents available for the treatment of T2D, only 40% of people with diabetes achieve optimal glycemic control [157,158]. Increasingly, glucose-lowering agents, such as the sodium glucose-like transporter-2 inhibitors (SGLT2i) and glucose-like peptide-1 receptor agonists (GLP-1Ra), are recognized to have pleiotropic effects beyond glucose-lowering, including bodyweight reduction, blood pressure reduction, and cardiovascular protection [159,160]. SGLT-2 inhibitors have been shown to have renoprotective qualities, with the largest clinical trial CREDENCE demonstrating the reduced progression of renal impairment in people with established DKD [161]. The dapagliflozin CKD study also demonstrated renoprotection in patients with DKD and non-diabetic kidney disease [162]. Treatments that wholistically improve glycemia, reduce body weight and blood pressure, and offer cardiorenal protection to patients with T2D are clearly the most desirable for the patient, clinician, and health system. 

Manipulating the gut microbiota may provide an additional treatment to prevent or treat T2D and DKD [163,164]. Diet is the most influential short- and long-term modulator affecting the gut microbiota. Dietary fibers are widely recognized as an important part of a healthy diet. However, all fibers have not proven to be equal [165]. Recent research has indicated that whole-plant fibers from growing vegetable matter contain a number of important micronutrients, whereas highly processed fibers or fibers from seed coats have reduced nutritional value [166]. Sugarcane bioflavonoids, for example, have been shown to reduce the postprandial glycemic response to high glycemic index starchy foods [167]. Interestingly, recent reports have demonstrated superior benefits from antioxidants found in natural fiber sources compared to purified supplemental antioxidants [168,169]. 

Prebiotics are dietary supplements that contain fermentable fibers resulting in a health benefit to their host. The most common form of these is non-absorbable dietary fiber. They cannot be hydrolyzed or absorbed by the gastrointestinal tract, but rather they are selectively fermented by intestinal and colonic bacteria. It is reported that dietary supplementation of prebiotics positively modulates and normalizes the microbiota by eliminating pathogens and promoting the growth of beneficial microorganisms [170,171]. Prebiotics can also improve the function of the mucosal barrier and mitigate immune reactions in patients with irritable bowel syndrome [170,172,173,174,175]. Modulating the microbiota has shown beneficial effects in patients with obesity and T2D [9]. Prebiotics can also significantly lower fasting blood sugar in patients with T2D [176]. The prebiotic inulin has been shown to reduce adiposity, cholesterol and enhance glucose metabolism in wild-type mice with diet-induced obesity [177]. Diabetic rats receiving inulin had decreased insulin resistance, fasting blood glucose and serum insulin levels, and increased fasting serum GLP-1 levels [178]. In human studies, inulin intake can increase rates of SCFAs in overweight-obese men as it improves fat oxidation and metabolism [179]. 

Probiotics are live microorganisms introduced into the gut for potential beneficial effects by promoting the growth of healthy bacteria. Dietary supplementation with the probiotics *Lactobacillus rhamnosus*, *Lactobacillus paracasei*, or *Bifidobacterium animalis* diminished the obesogenic effects of a high-fat diet in mice and reduced weight gain. Furthermore, probiotics may assist with glucose-insulin equilibrium as glucose levels were quicker to normalize in mice who received probiotics following an oral glucose bolus [180]. However, *Lactobacillus fermentum*, *Lactobacillus acidophilus*, and *Lactobacillus ingluviei* were associated with weight gain [181]. The outcomes of human studies are mixed. A meta-analysis study showed that the effect of *Lactobacillus*-containing probiotics also varies based on the species used. A significant weight gain was demonstrated in humans administered with *Lactobacillus acidophilus*, as an example, while *Lactobacillus gasseri* was associated with weight loss in obese humans [181]. In addition, when human fecal samples were studied, obesity was associated with higher levels of *Lactobacillus reuteri* regardless of the probiotic use [182].

Complementary medicines and natural healthcare supplements are widely embraced by the global community. Almost four out of ten American adults indicated that they used complementary medicines [183,184]. More studies are needed to explore the beneficial effects of prebiotic and probiotic use in the prevention and complementary treatment of DKD.

## 8. Fecal Microbiota Transplantation

There is increasing interest in fecal microbiota transplantation (FMT) as a treatment for various disease states, with the strongest evidence occurring for people with *Clostridium difficile*-induced colitis [185,186]. Conceptually, FMT involves infusion of the gut microbiota from a healthy subject to an unhealthy subject, altering their gut microbiota and influencing the disease state. Transfer of intestinal microbiota from a lean individual to a person with a metabolic syndrome was shown to significantly alter the intestinal microbiota in favor of the increased presence of butyrate-producer *Eubacterium hallii*, conferring a significant improvement in peripheral insulin sensitivity 6 weeks after the gut microbiota transplant [187]. Improved hepatic insulin sensitivity was also observed despite no change in dietary composition, resting energy expenditure, or hormonal changes. Using an obstructive sleep apnea–induced hypertensive model, which is known to decrease kidney function, Durgan et al. demonstrated that transplant of dysbiotic caecal contents from hypertensive to normotensive rats caused hypertension in the recipient [158], supporting the role of dysregulated gut microbiota in CKD. To our knowledge, FMT has not been undertaken in patients with DKD.

## 9. Conclusions

The association between gut microbiota and diabetes, obesity, and kidney disease have been well established. The increasing evidence for the association between gut dysbiosis and DKD confirms the gut-kidney interrelationship, yet the causal role of gut dysbiosis and DKD development and progression remains less clear. Nonetheless, effective natural-based therapies that can improve glycemic control, provide favorable metabolic benefits, and prevent diabetes-related complications are increasingly sought. Dietary manipulation, prebiotics, and probiotics are widely acceptable to patients in light of their general safety, low regulatory constraints, and quick route to market. Hence, manipulation of the microbiota, whether by fibers, prebiotics, probiotics, complementary medicines, or fecal microbiota transplant may offer a natural and safe addition to the treatment armamentarium for patients with diabetes and kidney-related disease.

## Figures and Tables

**Table 1:** Animal studies of the gut microbiota in T1D.

Reference	Authors	Year of Study	Animal Model	Study Findings
[74]	Wu et al.	2021	NOD/ltj T1D mice vs. ICR mice	Decreased microbiota diversity and community richness were shown in animals before the onset of T1D. T1D was associated with increased Firmicutes, Proteobacteria, and Deferribacteres phyla abundance. *Coprococcus 2*, *Lachnoclostridium_5*, and *Lachnospariceae_FCS020* genera (Firmicutes Phylum) were dominant in T1D, and their levels positively correlated with blood neutrophil ratios.
[75]	Ma et al.	2020	Streptozotocin-induced T1D rats vs. control	Firmicutes and Bacteroidetes are the dominant phyla in T1D rats. Pathogenic bacteria are abundant in T1D rats, while beneficial bacteria are reduced compared to control. *Butyricicoccus* and *Allobaculum* produce SCFAs and protect intestinal barrier function. Imbalance of gut microbiota causes reduction of SCFAs and intestinal inflammation.
[76]	Prasad et al.	2019	Angiotensin-converting enzyme 2 (ACE2) deficient T1D Akita mice vs. control	Increased Firmicutes and Bacteroidetes in the gut of ACE2^−/−^Akita mice ACE2 loss induced gut barrier permeability. Dysbiosis in the gut promoted the development of diabetic nephropathy.
[77]	Patterson et al.	2015	Streptozotocin (STZ) induced T1D in Sprague–Dawley rats (over time) vs. control	Animals with T1D have decreased microbial diversity and differential expression of microbiota. T1D onset was associated with an increase in Bacteriodetes: Firmicutes ratio. Increased lactic acid-producing bacteria (*Bifidobacterium* and *Lactobacillus*) was associated with late-stage T1D progression.
[78]	Hara et al.	2012	Virus-induced T1D rats vs. control	Virus-induced T1D animals expressed increased levels of *Bifibacterium* and *Clostridium* species and were reduced following antibiotic administration.
[79]	Roesch et al.	2009	Bio-breeding diabetes-prone (BB-DP) vs. bio-breeding diabetes-resistant (BB-DR) rats	BB-DR rats had more *Lactobacillus* and *Bifidobacterium* genera vs. BB-DP. Increased levels of 9 genera were also demonstrated in BB-DP rats in correlation with the onset of diabetes.
[80]	Brugman et al.	2006	Diabetic BB-DP rats before and after the onset of diabetes in the presence and absence of antibiotics	Rats protected from diabetes had a lower amount of *Bacteroides* species compared to rats with high levels. Modulation of microbiota by antibiotics reduced the incidence of T1D and delayed its onset.

**Table 2:** Human studies of the gut microbiota in T1D.

Reference	Authors	Year of Study	Number of Study Participants	Study Findings
[66]	Demirci et al.	2020	53 T1D vs. 53 healthy participants (Turkish population)	Firmicutes: Bacteroidetes ratio was decreased in T1D vs. healthy control. This correlated with TLR4 and TLR2 levels.
[67]	Huang et al.	2018	12 T1D vs. 10 healthy	T1D patients were characterized by decreased Firmicutes: Bacteroidetes ratios. HbA1c negatively correlated with *Faecalibacterium* levels. There was a positive correlation between *Bacteroides* levels and the presence of anti-islet cell autoantibodies.
[81]	Gürsoy et al.	2018	42 newly diagnosed T1D vs. 42 healthy	Increased levels of intestinal *Candida albicans* species at the time of diagnosis of T1D patients showing that normal intestinal microbiota is impaired.
[72]	Leiva-Gea et al.	2018	15 T1D, and 15 maturity-onset diabetes of the young 2 (MODY2) vs. 13 healthy	Decreased microbial diversity and increased gut permeability in the T1D group compared to other groups. Increased levels of *Bacteroides*, *Veillonella*, *Ruminococcus*, *Blautia*, and *Streptococcus* genera and reduced levels of *Bifidobacterium*, *Roseburia*, *Lachnospira*, and *Faecalibacterium* in the T1D group compared to control and patients with MODY2 (other non-autoimmune diabetes).
[71]	Higuchi et al.	2018	20 T1D vs. 28 healthy (Brazilian population)	Gram-negatives *Bacteroides* (*vulgatus*, *xylanisolvens*, and *rodentium*) and *Prevotella Copri* were more prevalent in patients with T1D in association with increased IL-6 and poor glycemic control.
[73]	Pellegrini et al.	2017	19 T1D vs. 16 healthy (Italian population)	Firmicutes: Bacteroidetes ratio was increased in the T1D group, while Bacteroidetes and Proteobacteria phyla levels were reduced. Specific correlation was uniquely demonstrated between the microbiota and 7 inflammatory genes expressed in patients with T1D.
[82]	Pinto et al.	2016	3 T1D vs. 3 healthy children	*Bacteroides* and *Clostridium* clusters XVa and IV were abundant in children with T1D. *Bifidobacterium* spp. were abundant in the healthy group. Identified 26 more abundant bacterial proteins in T1D and 21 more abundant proteins in healthy children. The specific protein functions of those genes were documented.
[83]	de Goffau et al.	2014	28 children (1-5 years old) with new-onset T1D vs. 27 healthy	Healthy children > 2.9 years had increased levels of *Clostridium* clusters IV and XIVa vs. T1D children or children < 2.9 years of age. Bacilli class (Streptococci) and Bacteroidetes phylum were higher in T1D children < 2.9 years vs. healthy, whereas healthy children had more levels of butyrate-producing species (*Clostridium* clusters IV and XIVa).
[84]	Soyucen et al.	2014	35 newly diagnosed T1D vs. 35 healthy	Reduction in the levels of beneficial anaerobic bacteria (*Bifidobacterium* genus) and increased *Candida albicans* and *Enterobacteriaceae* (other than *Escherichia coli*) species were demonstrated in newly diagnosed T1D vs. control.
[68]	Murri et al.	2013	16 children with T1D vs. 16 healthy	T1Ds had significant decrease in the ratio of Firmicutes: Bacteroidetes and reduced levels of the Blautia coccoides-Eubacterium rectal group (involved in butyrate production and maintenance of gut integrity). Increased *Clostridium*, *Bacteroides*, and *Veillonella* genera and reduced *Lactobacillus*, *Prevotella*, and *Bifidobacterium* genera were also demonstrated in children with T1D.
[70]	Giongo et al.	2011	4 children with T1D autoimmunity vs. 4 healthy	Firmicutes declined, and Bacteroidetes increased in the gut microbiota as children develop T1D. Reduced bacterial diversity was shown over time in autoimmune individuals. Autoimmune microbiota instability and the high ratio of Firmicutes: Bacteroidetes within the first 6 months after birth may be an indication of the future development of autoimmunity; before the detection of serum antibodies and might have potential in early diagnosis.
[69]	Brown et al.	2011	4 children with β-cell autoimmunity vs. 4 healthy	Firmicutes: Bacteroidetes ratio was reduced in autoimmune vs. healthy children. Lactate-producing bacteria (*Lactobacillus*, *Lactococcus*, *Streptococcus*, and *Bifidobacterium*) were less abundant in autoimmunity vs. control. Increased butyrate-producing bacteria (e.g., *Eubacterium*, *Subdoligranulum*, *Fusobacterium*, *Roseburia*, *Anaerostipes*, and *Faecalibacterium*) and mucin-degrading bacteria (*Prevotella* and *Akkermansia*) were shown in control vs. autoimmunity.

**Table 3:** Animal studies of the gut microbiota in T2D.

Reference	Authors	Year of Study	Subjects Number	Study Findings (Microbiota Changes in T2D vs. Control)
[85]	Kesh et al.	2020	T2D mice vs. control (both with pancreatic adenocarcinoma +/− chemotherapy)	*Bacteroides intestinalis* and *Lactobacillus murinus* species were more abundant in the control group. *Enterobacter cloacae* and *Bacteroides uniformis* species were specifically expressed in the T2D group. Dysbiosis in the microbiota seen in animals with T2D associated with increased resistance to chemotherapy.
[86]	Yin et al.	2020	T2D mice (induced by high-fat or high-sucrose-fat diet + STZ) vs. control	Bacteroidetes levels were reduced in high-fat diet-fed mice vs. high sucrose. Intestinal microbiota composition did not change after STZ, suggesting that the difference in metabolic phenotypes and gut microbiota was diet-related.
[87]	Grasset et al.	2017	T2D obese mice (fed HFD/ high-carbohydrate diet) and T2D mice (fed HFD/carbohydrate-free diet) compared to control (on chow diet)	Bacteroidales, Burkholderiales, Clostridiales, and TM7 (Saccharibacteria) orders were increased in T2D vs. control. Increased *Porphyromonadaceae*, *Clostridiaceae*, *Peptostreptococcaceae*, *Burkolderiaceae*, and TM7 families and reduced frequency of *Lactobacillaceae* in the T2D group was associated with Glucagon-like peptide 1 ( GLP-1) resistance. The gut-brain axis is impaired in T2D mice, and this can prevent beta cell sensitivity to GLP-1. Eubiotic microbiota required for GLP-1 sensitivity.
[88]	Everard et al.	2013	ob/ob mice and HFD-fed mice, with and without prebiotics vs. lean control mice	The presence of Bacterium inversely correlated with body weight. *Akkermansia (A) muciniphila* is reduced in obese mice and mice with T2D. *A. muciniphila* administration reversed obesity-related metabolic disease, increased the levels of endocannabinoids, and controlled inflammation.

**Table 4:** Human studies of the gut microbiota in T2D.

Reference	Authors	Year of Study	Subjects Number	Study Findings (Microbiota Changes in T2D vs. Control)
[89]	Li et al.	2020	20 T2D patients vs. 40 healthy individuals from North China	Decreased gut microbe diversity. T2D had reduced levels of Butyrate-producing bacteria (*Bifidobacterium*, *Akkermansia*, *Faecalibacterium*) and increased levels of bacteria involved in chronic inflammation (*Dorea*). Abundance in *Dorea*’s levels could identify patients at high risk for T2D.
[90]	Chávez-Carbajal et al.	2020	217 pre-diabetic and diabetic patients with and without treatment vs. healthy individuals (Mexican subjects)	Reduced bacterial richness and diversity. Pre-diabetic and diabetic groups had specific predicted metabolic characteristics and gut bacteria.
[91]	Doumatey et al.	2020	98 T2D Nigerians vs. 193 healthy Nigerians	Increased Actinobacteria and Bacteroidetes phyla. Decreased Firmicutes phylum. *Clostridiaceae* and *Peptostreptococcaceaea* families were significantly reduced.
[92]	Sedighi et al.	2017	18 T2D vs. 18 healthy	*Bifidobacterium* genus was reduced while *Lactobacillus* was significantly increased.
[93]	Inoue et al.	2017	12 T2D vs. 10 healthy (Japanese population)	11 pathways were significantly enriched in the diabetic group, including insulin signaling, glycolysis, and glycogenesis pathways. Significant reduction in *Blautia* genus, negatively correlated with HbA1c and glucose levels.
[94]	Wu et al.	2010	16 T2D vs. 12 healthy	Reduction in species profiles and reduced levels of *Bifidobacterium* genus and *Bacteroides vulgatus.*
[95]	Larsen et al.	2010	18 T2D vs. 18 healthy	Bacteroidetes: Firmicutes ratio and *Bacteroides-Prevotella* to *Blautia coccoides -Eubacterium* rectal correlated positively with plasma glucose levels.
[96]	Bilen et al.	2007	66 T2D vs. 50 healthy	Conjunctival flora from patients with T2D had increased *Staphylococcus aureus* and *Staphylococcus epidermidis* compared to control.

**Table 5:** Animal studies of the gut microbiota in obesity.

Reference	Authors	Year of Study	Animal Model	Study Findings (Microbiota Changes in Obesity vs. Control)
[103]	Sang et al.	2021	Obese (HFD-fed mice) with and without *Ganoderma lucidum* vs. control (low-fat diet)	Obese (HFD-fed mice) had increased Firmicutes: Bacteroidetes ratio in the gut microbiota vs. control. Reduced *Bifidobacterium choerinum* and *Bacteroide chinchillae* levels. *Ganoderma lucidum* treatment increased *Allobacullum*, *Christensenella*, and *Bifidobacterium* and inhibited body weight increase and inflammation due to HFD in association with increased SCFAs levels and GPR43 activation.
[104]	Beckmann et al.	2021	Diet-induced obesity in rats (with and without telmisartan) vs. control	Increased Firmicutes: Bacteroidetes ratio in diet-induced obesity vs. control. Increased Blautia, *Allobaculum*, and *Parasutterella* levels. Transfer of stool from telmisartan-treated mice to obese mice attenuated the increase in body weight due to dietary-induced obesity.
[105]	Bagarolli et al.	2017	HFD-fed mice with and without probiotics vs. control	HFD increased Bacteroidetes and decreased the levels of Firmicutes and Actinobacteria phyla. HFD increased intestinal permeability. Probiotics reduced fat pad and weight gain and improved insulin resistance and glucose tolerance.
[106]	Lam et al.	2012	Obese (HFD-fed mice) vs. control (chow diet-fed mice)	Reduced *Lactobacillus* genus leading to increased inflammatory cytokines. Increased fecal *Oscillibacter* genus. *Lactobacillus* and *Oscillibacter* levels correlated with transepithelial resistance of the proximal colon.
[102]	Hildebrandt et al.	2009	Control mice (13-week chow diet) or Restin-like molecule (RELM)-β knockout mice switched to HFD for 21 weeks	Reduced Bacteroidetes and increased Firmicutes and Proteobacteria phyla associated with switch to HFD. HFD (independent of obesity) was mainly responsible for the observed changes in microbiota.
[107]	Turnbaugh et al.	2006	Genetically obese mice (ob/ob) vs. control	Germ-free mice colonized with obese microbiota led to increased total body fat

**Table 6:** Human studies of the gut microbiota in obesity.

Reference	Authors	Year of Study	Subjects	Study Findings for Obese vs. Control
[108]	Da Selva	2020	21 children with obesity/overweight vs. 30 healthy (Caribbean island of Trinidad)	Reduced alpha diversity was shown compared to lean children. Firmicutes was associated with overweight/obese children. *Bifidobacterium* was associated with healthy weight children.
[109]	Gao et al.	2018	71 obese and 22 overweight vs. 25 healthy	Reduced gut microbial diversity and reduced beneficial bacteria, e.g., *Faecalibacterium*, *Bifidobacterium*, and *Ruminococcaceae.* Increased levels of Proteobacteria (*Pseudomonas*, *Fusobacterium*, *Escherichia*, and *Shigella*).
[110]	Kalliomäki et al.	2008	25 obese vs. 24 normal children (prospective follow-up study from children 3 months to 7 years)	Children who had higher levels of *Bifidobacteria* maintained a normal weight over time, whereas children who had greater levels of *Staphylococcus aureus* became overweight.
[111]	Collado et al.	2008	18 overweight pregnant women vs. 36 normal-weight pregnant women (prospective follow-up study during pregnancy)	High Bacteroidetes concentrations before pregnancy were associated with excessive weight gain during pregnancy. BMI and mother’s weight correlated with concentrations of *Clostridium*, Bacteroidetes, and *Staphylococcus*. Microbial counts were increased in the third trimester of pregnancy compared to the first trimester.

**Table 7:** Animal studies of the gut microbiota in CKD.

Reference	Authors	Year of Study	Animal Species	Study Findings in Diseased Group vs. Control
[131]	Nishiyama et al.	2019	CKD mice (5/6 nephrectomy) vs. controls	Reduced *Lactobacillus*, *Oscillospira*, and unclassified *Ruminococcaceae* genera in mice with CKD while *Bifidobacterium*, *Turicibacter*, and *Allobaculum* genera were increased.
[132]	Yang et al.	2018	Adenine-induce CKD mice vs. control group (with and without prebiotic fiber)	Prebiotic supplementation reduced Clostridium, Erysipelotrichaceae, unclassified Lactobacillus, Allobaculum, Staphylococcus, and Dorea levels but had no effect on Ruminococcus, Blautia, and Coprobacillus.
[133]	Yang et al.	2015	Spontaneous hypertensive rats and angiotensin II-infused chronic hypertensive rats compared to controls	Decreased microbial diversity in hypertensive rats vs. controls. Firmicutes: Bacteroidetes ratio was increased, associated with reduced acetate and butyrate-producing bacteria.
[134]	Vaziri et al.	2013	CKD (5/6 nephrectomy rats) vs. control	Rats with chronic renal failure showed increased Betaproteobacteria class but decreased Actinobacteria, Proteobacteria phyla, *Lactobacillaceae*, *Prevotellaceae* families, Clostridia, Mollicutes, Bacilli, and Bacteroidiaclass.
[135]	Tanida et al.	2005	Hypertensive Wistar rats vs. healthy rats (administered with *Lactobacillus johnsonii* probiotic)	Administration of *Lactobacillus johnsonii* reduced blood pressure and might be due to altering renal sympathetic nerve activity.
[136]	Kawase et al.	2000	Rats fed fermented milk with *Streptococcus thermophilus*, *Lactobacillus casei*, or both vs. control	Atherogenic index, systolic blood pressure, and total serum cholesterol level were reduced.

**Table 8:** Studies of the gut microbiota in chronic kidney disease (CKD) in humans.

Reference	Authors	Year	Patient Group	Study Findings in Diseased Group vs. Control
[144]	Jiang et al.	2017	52 with ESKD vs. 60 healthy	Switch from *Prevotella* (enterotype 2) to *Bacteroides* (enterotype 1) was associated with a reduction of butyrate-producing bacteria.
[19]	Xu et al.	2017	32 CKD vs.32 healthy	Reduced bacterial diversity. The levels of opportunistic pathogens from gamma-proteobacteria were increased. However, beneficial microbes like *Coprococcus*, *Roseburia*, and *Ruminococcaceae* were decreased. Impaired renal function and gut microbiota dysbiosis increased plasma concentrations of trimethylamine-N-oxide involved in cardiovascular disease.
[145]	Salgado et al.	2016	Pediatric patients; 8 on peritoneal dialysis, 8 hemodialysis 10 post kidney transplants vs. 13 healthy	Lower bacterial species richness was demonstrated in peritoneal dialysis and post-transplant patients compared with healthy individuals and patients on hemodialysis.
[140]	Jiang et al.	2016	65 CKD (Stage 1–5) vs. 20 healthy	Reduced butyrate-producing bacteria *Faecalibacterium prausnitzii* and *Roseburia* spp. with a mild reduction in kidney function in comparison with healthy individuals.
[133]	Yang et al.	2015	7 Hypertensive patients vs. 10 healthy	Reduced bacterial diversity. Increased Firmicutes: Bacteroidetes ratio.
[146]	Wong et al.	2014	24 patients with ESKD undergoing hemodialysis vs. 12 healthy	Expansion in the bacterial families that possess uricase, urease, indole, and p-cresol–forming enzymes and a reduction in bacterial families with butyrate-forming capability compared with healthy individuals.
[134]	Vaziri et al.	2013	24 patients with ESKD undergoing hemodialysis vs. 12 healthy	Difference in the abundance of ca. 190 microbial operational taxonomic units (OTU) when the gut microbiota was compared to healthy controls.
[147]	I. Wang et al.	2012	29 patients with ESKD undergoing PD vs. 41 healthy	Abundances of Bifidobacterium and Lactobacillus spp. (B. Catenulatum, B. longum, B. bifidum, L. plantarum, and L. paracasei) were reduced compared with healthy individuals.
[148]	Wang et al.	2012	30 patients with ESKD not on dialysis vs. 10 healthy	Bacterial DNA detected in the blood of 20% of patients. Bacterial genera identified in the patient’s blood were overgrown in the guts of the same patients.
[141]	Ranganathan et al.	2009	13 patients with CKD (Stage 3 and 4)	Decreased levels of culturable anaerobic bacteria were shown in the feces of patients with CKD compared to healthy controls.
[142]	Fukuuchi et al.	2002	27 patients with chronic kidney failure and 20 patients with hemodialysis vs. 12 healthy controls	Culturable aerobic bacteria levels were increased in the feces of patients with CKD, not yet on dialysis, when compared with healthy adults.
[143]	Hida et al.	1996	ESKD patients 20 with hemodialysis vs. 12 healthy controls	Aerobic bacteria, including the *Enterococci* and *Enterobacteria* species, were increased in quantity in patients with ESKD compared with healthy controls.

## Data Availability

Not applicable.
